# Inertial Navigation System/Doppler Velocity Log (INS/DVL) Fusion with Partial DVL Measurements

**DOI:** 10.3390/s17020415

**Published:** 2017-02-22

**Authors:** Asaf Tal, Itzik Klein, Reuven Katz

**Affiliations:** 1 Faculty of Mechanical Engineering, Technion-Israel Institute of Technology, Haifa 3200003, Israel; reuvenk@technion.ac.il; 2 Rafael Advanced Defense Systems Ltd, Haifa 3102102, Israel; itzikkl@rafael.co.il

**Keywords:** Doppler velocity log/inertial navigation system (DVL/INS) fusion, loosely coupled, tightly coupled, autonomous underwater vehicle six degrees of freedom (AUV 6DOF) simulation, partial measurement

## Abstract

The Technion autonomous underwater vehicle (TAUV) is an ongoing project aiming to develop and produce a small AUV to carry on research missions, including payload dropping, and to demonstrate acoustic communication. Its navigation system is based on an inertial navigation system (INS) aided by a Doppler velocity log (DVL), magnetometer, and pressure sensor (PS). In many INSs, such as the one used in TAUV, only the velocity vector (provided by the DVL) can be used for aiding the INS, i.e., enabling only a loosely coupled integration approach. In cases of partial DVL measurements, such as failure to maintain bottom lock, the DVL cannot estimate the vehicle velocity. Thus, in partial DVL situations no velocity data can be integrated into the TAUV INS, and as a result its navigation solution will drift in time. To circumvent that problem, we propose a DVL-based vehicle velocity solution using the measured partial raw data of the DVL and additional information, thereby deriving an extended loosely coupled (ELC) approach. The implementation of the ELC approach requires only software modification. In addition, we present the TAUV six degrees of freedom (6DOF) simulation that includes all functional subsystems. Using this simulation, the proposed approach is evaluated and the benefit of using it is shown.

## 1. Introduction

Underwater operations attract great attention for environmental matters and potential resources, as well as scientific interest. Therefore, the need for underwater robotic systems has become more apparent [1]. With the emergence of inspection-class autonomous underwater vehicles (AUVs), navigation and navigational accuracy are becoming increasingly important in order for the AUV to complete its task. Without an operator in the loop, the vehicle must use sensors to determine its position, velocity and orientation. Most AUVs employ an inertial navigation system (INS) as their main navigation sensor [2,3,4]. This is for many reasons; one of which is that the INS is a standalone system that can provide all of the required navigation data: position, velocity and orientation. However, even with a high-grade INS, the navigation solution drifts in time due to measurement errors of its inertial sensors. Therefore, INSs are usually aided by other external sensors or data such as the Doppler velocity log (DVL) for velocity, magnetometers for heading and depth/pressure sensor for altitude [5,6]. For positioning, global positioning system (GPS) is commonly used in land or air vehicles, yet it cannot be used underwater [7,8] since the electromagnetic signals decay very quickly in the water. A common method for overcoming the localization problem of underwater vehicles is to use acoustic positioning methods such as long baseline (LBL) or ultra-short baseline (USBL) [9]. One drawback of such approaches is the need for a priori deployment of beacons. An alternative method for obtaining localization of underwater vehicles is geophysical navigation which uses physical features of the AUV’s environment to produce an estimate of the location of the AUV [10,11].

A team at the Technion–Israel Institute of Technology is currently developing an AUV named the Technion autonomous underwater vehicle (TAUV). The TAUV project goal is to develop and to produce a small autonomous underwater vehicle, which will serve as a technology demonstrator and a platform for various research programs. The TAUV diameter is 300 mm and its length is 3000 mm. The TAUV has four rudders installed in an “x” configuration, and its maximum operation depth is 200 m. The TAUV navigation system based on an INS aided by a DVL, magnetometer, pressure sensor (PS), and GPS when available [12]. When submerged, the main aiding sensor is the DVL which provides velocity measurements that can help estimate, in addition to the vehicle velocity, some of the orientation and inertial sensor error states depending on the vehicle dynamics [13,14].

**Figure 1:**
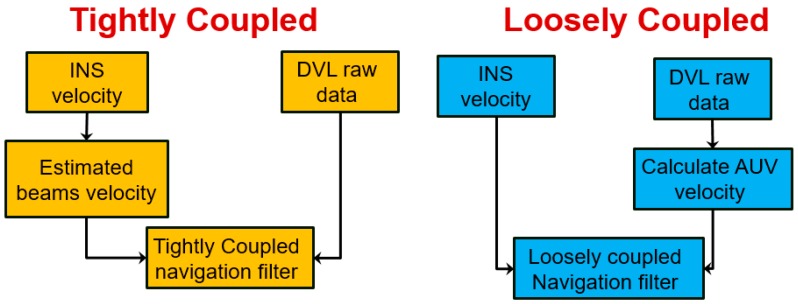
Scheme of loosely coupled and tightly coupled approaches for INS/DVL fusion. AUV: Autonomous underwater vehicle; DVL: Doppler velocity log; INS: inertial navigation system.

There are two main approaches for sensor fusion between INS and other sensors: (1) loosely coupled (LC); and (2) tightly coupled (TC) [15]. We shall refer here to INS/DVL fusion, but the underlying principles are the same for other sensors as well. A top-level block diagram of LC and TC approaches is presented in Figure 1. The raw data of the DVL is the relative velocity in each beam direction. In the LC approach, using parameter estimation the DVL raw data is used to calculate the vehicle velocity, which in turn is compared with its INS counterpart in the navigation filter. The advantage of this method is the simplicity of integration and the ability to combine any off-the-shelf INS with any DVL. However, in order for the DVL to calculate vehicle velocity, it must operate in bottom lock, which refers to the condition when a sufficient number of beam measurements (at least three) are available. In the TC approach, the DVL raw data is directly used in the navigation filter. That is, each beam measurement of the DVL is compared with its calculated INS counterpart and independently integrated into the navigation filter. Therefore, there is no need for a bottom lock stage, and aiding may be applied even with a single beam measurement.

The selected TAUV INS can only receive from the DVL an external velocity vector measurement [16]. Therefore, INS/DVL fusion with the TC approach is not possible, and thus the LC approach must be used. In some situations—such as operation in close proximity to the seafloor, or when experiencing extreme tilt angle or beam malfunction—one or more of the DVL beams may not provide the reflection required for determining velocity [17]. In such cases of partial beam measurements, the DVL fails to maintain bottom lock and is unable to estimate the AUV velocity. Thus, the TAUV INS navigation solution will drift in time.

In this paper, we propose the extended loosely coupled (ELC) approach for calculating vehicle velocity when only partial DVL measurements are available. This is made possible by using the partial measured raw data from the DVL combined with additional information. This calculated vehicle velocity is fed back to the TAUV INS and used for aiding the INS, as in the regular LC approach.

Related approaches in similar situations where partial measurements are available can be found in the literature for INS/GPS fusion or LBL positioning. In [18], a single beacon measurement and two virtual ranging measurements were used to triangulate and solve the vehicle position with the LBL method. Another method is to utilize the knowledge of the vehicle dynamics under a specific scenario. For example, in [19] the authors utilized the vehicle dynamics in a straight trajectory scenario to improve navigation performance with low-cost GPS. In [20] the construction of virtual GPS satellites was suggested in order to facilitate GPS receiver position and velocity solutions in cases of partial GPS availability, thereby enabling the fusion of GPS/INS in the LC approach.

In order to examine and demonstrate the improvement of the navigation performance under the ELC approach, we developed the TAUV six degrees of freedom (6DOF) simulation, which includes all functional sub-systems. This simulation consists of the AUV guidance, navigation and control subsystems and uses a complete hydrodynamic model. Simulation results show great improvement in the navigation performance using the proposed approach compared to the standalone TAUV INS solution.

The rest of the paper is organized as follows: Section 2 describes the TAUV 6DOF simulation and in particular its navigation system. In Section 3 the ELC approach is derived. In Section 4 6DOF simulation results are presented to demonstrate the effectiveness of the partial measurements approach on the TAUV navigation system. Finally, Section 5 provides the conclusions.

## 2. TAUV 6DOF Simulation

**Figure 2:**
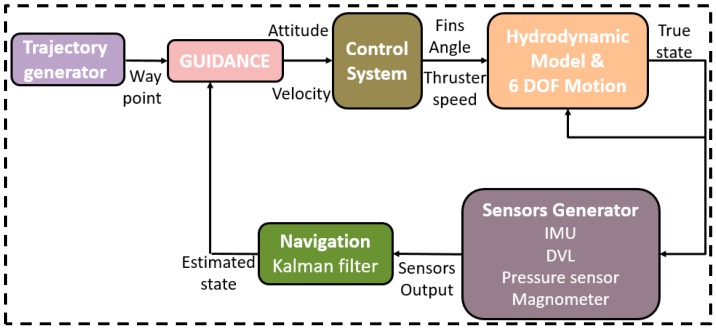
Technion autonomous underwater vehicle six degrees of freedom (TAUV 6 DOF) simulation layout. IMU: inertial measurement unit.

A top-level block diagram of the TAUV 6DOF simulation with its main subsystems is presented in Figure 2. The purpose of each subsystem is discussed next.

**Hydrodynamic and Motion block**: This model calculates the forces and moments applied on the TAUV for the 6DOF rigid body motion model. The motion model solves the vehicle kinematics to produce the true values of the vehicle’s position, velocity, attitude, and acceleration. The model contains a complete hydrodynamic model derived for the TAUV.

**Control system block**: The control system is used to stabilize the vehicle attitude by determining the angles of the four TAUV servos. The vehicle velocity is stabilized by controlling the thruster rotation speed. The TAUV control system contains four proportional integrative derivative (PID) controllers: three for the attitude control and one for the velocity control.

**Guidance system block**: The guidance laws in the TAUV are based on target tracking methods [21]. This subsystem determines the vehicle steering command and the desired velocity in order to follow the AUV’s desired trajectory.

**Sensors generator block**: The block calculates sensors’ outputs, including their errors, by statistical means. The sensors are: accelerometers, gyros, DVL, PS, magnetometer, and GPS.

**Navigation block**: The navigation block represents the navigation system of the TAUV, including the navigation equations and navigation filter. The TAUV navigation system is presented in detail in the following section.

### 2.1. TAUV Navigation System

**Figure 3:**
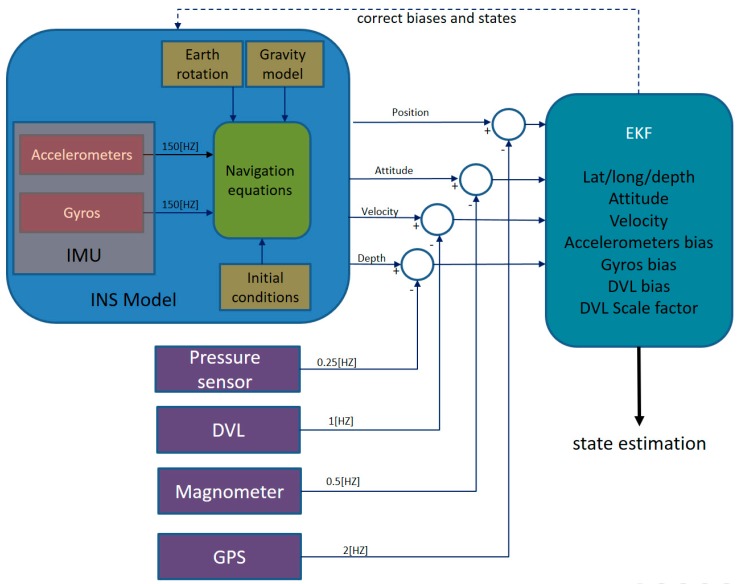
Top level diagram of the TAUV navigation system. EKF: extended Kalman filter. GPS: global positioning system.

In Figure 3, a top-level block diagram of the TAUV navigation system is shown. The accelerometer and gyro measurements are integrated into the INS system in order to produce its standalone solution for position, velocity, and attitude. The extended Kalman filter (EKF) [22,23,24] fuses the INS with measurements from the other sensors: the magnetometer, PS, DVL and GPS. By comparing the INS state calculation and aiding the sensor measurements, the EKF estimate corrects the error state vector comprising the INS states and inertial sensor error terms. Notice that each aiding sensor operates at a different sampling rate, yet in each time instance that a measurement arrives, it is fused with the INS.

**Figure 4:**
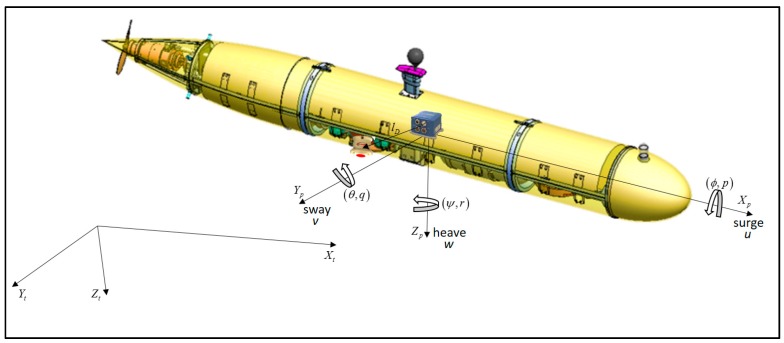
Platform frame and tangent frame in respect to the TAUV.

The navigation equations and the EKF are expressed in a coordinate frame, known as the platform frame. The platform frame is located at the center of buoyancy of the vehicle. The navigation frame is determined by the INS frame. For simplicity of the dynamics equations, we assume that the platform frame and the INS frame are located at the same point at the AUV and with identical orientation. The TAUV platform frame and the tangent frame are represented in Figure 4. The vehicle velocities (u,v,w) are expressed in the platform frame, and the vehicle attitudes expressed in the tangent frame by the roll, pitch, and yaw (ϕ,θ,ψ) Euler angles.

#### 2.1.1. Navigation Filter

An error state EKF is implemented in the TAUV navigation system. The error state vector δx is defined by the difference between the true state vector and Kalman filter estimate and expressed by [17]:

(1) $$\delta x_{k}=x_{k}-{\hat{x}}_{k}$$

where k is the time step. The standard INS error state vector is in accordance with [22]:

(2) $$\delta x={\left[\begin{array}{ccccc} {\left(\delta r_{t/p}^{t}\right)}^{T} & \delta \rho^{T} & {\left(\delta v_{t/p}^{p}\right)}^{T} & \delta b_{a}^{T} & \delta b_{g}^{T} \end{array}\right]}^{T}\in {\mathbb{R}}^{15\;\times \;1}$$

where $\delta r_{t/p}^{t}$ is the position error vector expressed in the tangent frame; the vector δρ is the orientation error also referred to as the frame misalignment error; $\delta v_{t/p}^{p}$ is the velocity error vector expressed in the platform frame; $\delta b_{a}$ is the accelerometer biases error expressed in the platform frame; and $\delta b_{g}$ is the gyro biases error expressed in the platform frame. The INS error state model has the following form:

(3) $$\delta \dot{x}\left(t\right)=F_{INS}\left(t\right)\delta x+G_{INS}(t)w_{INS}\left(t\right)$$

where $F_{INS}\left(t\right)$ is the matrix that relates the error state to the dynamic state [17]:

(4) $$F_{INS}=\left[\begin{array}{ccccc} 0_{3\times 3} & -\left[{\hat{R}}_{p}^{t}{\hat{v}}_{t/p}^{p}\times \right] & {\hat{R}}_{p}^{t} & 0_{3\times 3} & 0_{3\times 3}\\ 0_{3\times 3} & -{\hat{\Omega }}_{i/e}^{t} & 0_{3\times 3} & 0_{3\times 3} & -{\hat{R}}_{p}^{t}\\ 0_{3\times 3} & F_{32} & F_{33} & -I_{3} & -\left[{\hat{v}}_{t/p}^{p}\times \right]\\ 0_{3\times 3} & 0_{3\times 3} & 0_{3\times 3} & 0_{3\times 3} & 0_{3\times 3}\\ 0_{3\times 3} & 0_{3\times 3} & 0_{3\times 3} & 0_{3\times 3} & 0_{3\times 3} \end{array}\right]\in {\mathbb{R}}^{15\;\times \;15}$$

${\hat{R}}_{p}^{t}$ is the estimated transformation matrix from platform frame to tangent frame, and ${\hat{\Omega }}_{i/e}^{t}$ is a skew symmetric matrix of the earth’s rotation rate expressed in the tangent frame. The expressions for $F_{32}$, $F_{33}$ can be found for example in [17]. The matrix $G_{INS}\left(t\right)$ relating the error state to the noise process is defined by:

(5) $$G_{INS}\left(t\right)=\left[\begin{array}{cc} \begin{array}{cc} 0_{3\times 3} & 0_{3\times 3}\\ 0_{3\times 3} & -{\hat{R}}_{p}^{t}\\ -I_{3} & -\left[{\hat{v}}_{t/p}^{p}\times \right]\\ 0_{3\times 3} & 0_{3\times 3}\\ 0_{3\times 3} & 0_{3\times 3} \end{array} & \begin{array}{cc} 0_{3\times 3} & 0_{3\times 3}\\ 0_{3\times 3} & 0_{3\times 3}\\ 0_{3\times 3} & 0_{3\times 3}\\ I_{3} & 0_{3\times 3}\\ 0_{3\times 3} & I_{3} \end{array} \end{array}\right]\in {\mathbb{R}}^{15\;\times \;12}$$

The process noises vector is defined by:

(6) $$w_{INS}\left(t\right)={\left[\begin{array}{cc} \begin{array}{cc} w_{a}^{T} & w_{g}^{T} \end{array} & \begin{array}{cc} w_{ba}^{T} & w_{bg}^{T} \end{array} \end{array}\right]}^{T}\in {\mathbb{R}}^{12\;\times \;1}$$

where $w_{a}$, $w_{g}$ are the accelerometers and gyros noise vectors are modeled as zero-mean white Gaussian processes, $w_{ba}$ is the noise vector of the accelerometer biases modeled as random walk and $w_{bg}$ is the noise vector of the gyro biases modeled as random walk.

In the linear case, the measurement model is:

(7) $$\delta y=H\times \delta x_{k}+n_{w}$$

where $n_{w}$ is zero-mean Gaussian white noise. The matrix H relates the measurement to the error state and is known as the measurement matrix. For each aiding sensor and for each measurement, a measurement rejection algorithm is applied, by examining if the data is within range of three standard deviations compared to the INS estimation.

In the presented navigation system model, we augment the INS state vector from Equation (2) with five additional error states of the DVL: four to model the DVL biases of each beam, and one for common DVL scale factor error. Thus, the augmented error state vector:

(8) $$\delta x={\left[\begin{array}{cc} \begin{array}{cccccc} {\left(\delta r_{t/p}^{t}\right)}^{T} & \delta \rho^{T} & {\left(\delta v_{t/p}^{p}\right)}^{T} & \delta b_{a}^{T} & \delta b_{g}^{T} & \delta b_{DVL}^{T} \end{array} & \delta SF_{DVL} \end{array}\right]}^{T}\in {\mathbb{R}}^{20\;\times \;1}$$

where $\delta b_{DVL}$ is the DVL biases error expressed in the DVL frame and $SF_{DVL}$ is the DVL scale factor error. The corresponding dynamics matrix *F* is given by:

(9) $$F\left(t\right)=\left[\begin{array}{cc} F_{INS} & 0_{15\times 5}\\ 0_{5\times 15} & 0_{5\times 5} \end{array}\right]\in {\mathbb{R}}^{20\times 20}$$

The corresponding noise process matrix *G* is expressed via:

(10) $$G\left(t\right)=\left[\begin{array}{cc} G_{INS} & 0_{15\times 5}\\ 0_{5\times 12} & I_{5} \end{array}\right]\in {\mathbb{R}}^{20\times 17}$$

where the filter process noises vector is modified via:

(11) $$w\left(t\right)={\left[\begin{array}{cc} \begin{array}{cc} \begin{array}{cc} w_{a}^{T} & w_{g}^{T} \end{array} & \begin{array}{cc} w_{ba}^{T} & w_{bg}^{T} \end{array} \end{array} & \begin{array}{cc} w_{bDVL}^{T} & w_{sfDVL}^{T} \end{array} \end{array}\right]}^{T}\in {\mathbb{R}}^{17\;\times \;1}$$

where $w_{bDVL}$ is a noise vector modeled as random walk of the DVL biases, and $w_{sfDVL}$ is zero-mean Gaussian white noise of the DVL scale factor.

#### 2.1.2. Doppler Solution

The DVL measures the Doppler frequency shift for each depth cell and each beam and then computes the component of relative flow velocity in the direction of each acoustic beam. Based on [25], the relative velocity of each beam can be assumed as:

(12) $$V_{rel}=\left(F_{D}+b_{F_{D}}+n_{F_{D}}\right)\frac{C\left(1+SF_{c}\right)}{2F_{s}}1000$$

where $F_{D}$ is the Doppler frequency shift, $b_{F_{D}}$ is the frequency shift bias, $n_{F_{D}}$ is the frequency shift noise, C is the velocity of sound in water at transducer face, $F_{s}$ is the transmitted acoustic frequency, $SF_{c}$ is the scale factor error of the velocity of sound in water, and $n_{F_{D}}$ is zero-mean Gaussian white noise. The scale factor is due to the variation of the velocity of sound under different temperatures and salinity level of the water. From Equation (12) we can observe that the DVL measurement model has three main errors: (1) bias error (2) scale factor error and (3) white noise.

**Figure 5:**
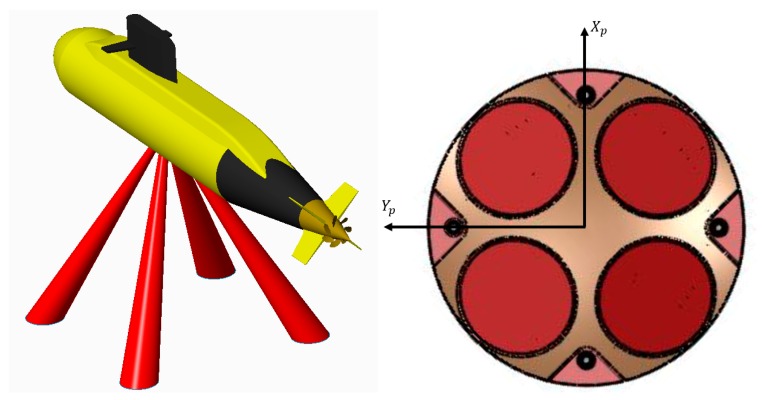
DVL orientation setup in the TAUV platform frame.

The DVL assembly in the TAUV is constructed in an “x” configuration relative to the platform frame (“Janus Doppler configuration”), as shown in Figure 5. Generally, each DVL beam direction in the vehicle frame is defined by:

(13) $$b_{i}=\left[\begin{array}{c} c{\tilde{\psi }}_{i}s\tilde{\theta }\\ s{\tilde{\psi }}_{i}s\tilde{\theta }\\ c\tilde{\theta } \end{array}\right]$$

where $b_{i}$ represents the direction of each transducer/beam of the DVL expressed in the platform frame, for i = 1,2,3,4, $\tilde{\theta }$ is the pitch angle (relative to platform frame) of each DVL beam, and ${\tilde{\psi }}_{i}$ is the yaw angle (relative to platform frame) of each DVL beam for i = 1,2,3,4. In the TAUV DVL, $\tilde{\theta }$ is equal to 20°.

The expression for yaw angle of each beam direction in the TAUV can be written as:

(14) $${\tilde{\psi }}_{i}=\left(i-1\right)90{}^{\circ}+45{}^{\circ},\;i=1,2,3,4$$

The DVL beam velocity is modeled as:

(15) $${\tilde{y}}_{v_{i}}=\left[v_{t/p}^{p}\left(1+SF_{DVL}\right)+\omega_{t/p}^{p}\times l_{D_{i}}\right]\times b_{i}+n_{v_{i}}+b_{DVL,i}$$

where $l_{D_{i}}$ is the position vector of each transducer expressed in the platform frame, $\omega_{t/p}^{p}$ is the angular rate vector of the platform expressed in the platform frame, $b_{DVL}$ is a 4 × 1 vector representing the bias of each beam, $SF_{DVL}$ is the scale factor error, and $n_{v_{i}}$ is measurement noise. Let *A* be a matrix contacting all beam direction vectors:

(16) $$A=\left[\begin{array}{c} b_{1}^{T}\\ b_{2}^{T}\\ b_{3}^{T}\\ b_{4}^{T} \end{array}\right]$$

Thus, by using Equation (15), the DVL-based velocity measurement of the vehicle can be expressed by:

(17) $${\tilde{v}}_{t/p}^{p}={\tilde{y}}_{v_{i}}{\left(A^{T}A\right)}^{-1}A^{T}$$

Note that the velocity solution in Equation (17) is based on four beams, of which one beam is for redundancy due to the beam configuration. Therefore, in practice only three beams are enough to calculate the vehicle velocity.

#### 2.1.3. DVL Fusion

In the LC fusion technique, the DVL output to the navigation filter is the platform velocity (Equation (17)). The corresponding measurement matrix is:

(18) $$H=\left[\begin{array}{ccccccc} 0 & 0 & I & 0 & 0 & 0 & 0 \end{array}\right]\in {\mathbb{R}}^{20\times 3}$$

and the velocity measurement noise covariance matrix is:

(19) $$R_{vel}={\left(A^{T}WA\right)}^{-1}\in {\mathbb{R}}^{3\times 3}$$

where $W=R_{DVL}^{-1}$ and $R_{DVL}$ is the DVL measurement noise covariance matrix [17], defined as:

(20) $$R_{DVL}=b_{i}^{T}\left[l_{D_{i}}\times \right]\sigma_{g}^{2}I{\left[l_{D_{i}}\times \right]}^{T}b_{i}+\sigma_{v}^{2}\in {\mathbb{R}}^{4\times 4}$$

In the case of small-level arm $l_{D_{i}}$, Equation (20) can be well approximated as:

(21) $$R_{DVL}\approx diag\left(\sigma_{v1}^{2},\sigma_{v2}^{2},\sigma_{v3}^{2},\sigma_{v4}^{2}\right)\in {\mathbb{R}}^{4\times 4}$$

where $\sigma_{v_{i}}$ is the velocity variance for each beam measurement for i = 1,2,3,4. The variance value of each beam is taken from the DVL spec [26] or calculated in a lab test.

In the TC fusion technique, the DVL raw data is used to aid the INS. That is, each transducer measurement is fused with its INS counterpart (no need to calculate the DVL vehicle velocity, Equation (17)) and the measurement matrix [17] is defined as:

(22) $$H_{TC}=\left[\begin{array}{ccc} \begin{array}{ccccc} 0 & 0 & A\left(1+S\hat{F}\right) & 0 & A\left[l_{D_{i}}\times \right] \end{array} & I & A\times {\hat{v}}_{t/p}^{p} \end{array}\right]\in {\mathbb{R}}^{20\times 4}$$

The measurement noise covariance matrix is taken from Equation (21) for each beam.

## 3. INS/DVL Fusion with the ELC Approach

As mentioned in Section 1, in some situations several or all of the DVL beams do not provide reflection. With no reflection, the beam cannot provide the relative velocity of the AUV Equation (15). In cases where only one beam is not available, the DVL can still calculate the vehicle velocity Equation (17) but without any redundant data. If two beams are not available, the DVL cannot calculate the vehicle velocity. In this case, no velocity aiding is provided to the TAUV INS.

**Figure 6:**
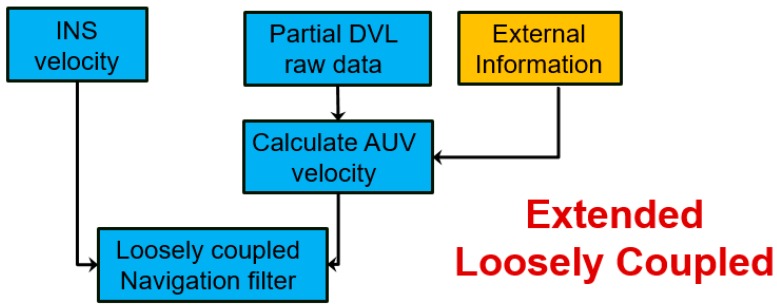
Extended loosely coupled (ELC) shame for utilizing partial measurements from the DVL.

In this section, we present the ELC approach, with four different methods for utilizing the partial measurements from the DVL (instead of not using them at all) together with external information to construct the DVL based velocity estimate. This velocity is used in the TAUV navigation filter, as illustrated in Figure 6. The four ELC methods differ in the external information they employ as elaborated in the following subsections.

### 3.1. Virtual Beam

This method utilizes the raw data from the DVL and the filter prediction of the velocity error in order to solve for the vehicle velocity. Without the loss of generality, we assume that beams #3 and #4 are not available. Let the matrix $A_{s}$ relate the velocity in the platform frame to the velocity in each beam direction. So, for small value of lever arm, following Equation (17) the velocities in beam directions (#1, #2, and #3) are:

(23) $$y_{v}=A_{s}\times v_{t/p}^{p}\;A_{s}={\left[\begin{array}{ccc} b_{1}^{T} & b_{2}^{T} & b_{3}^{T} \end{array}\right]}^{T}$$

From Equation (23), the value of the third beam may be found as:

(24) $$y_{v3}=b_{3}^{T}\;\left[v_{t/p}^{p}\right]\;y_{v3}\approx b_{3}^{T}{\left[\begin{array}{ccc} {\hat{u}}_{k-1} & {\hat{v}}_{k-1} & {\hat{w}}_{k-1} \end{array}\right]}^{T}$$

where $v_{t/p}^{p}$ is taken from the last INS step. In this method we estimate only the value of the third beam. Generally, the value of the fourth beam can be estimated instead. Plugging Equation (24) into Equation (23) we solve the unknown vehicle velocity vector at time k via:

(25) $$\left[\begin{array}{c} u_{k}\\ v_{k}\\ w_{k} \end{array}\right]={\left[A\right]}^{-1}\left[\begin{array}{c} y_{v1}\\ y_{v2}\\ y_{v3} \end{array}\right]$$

It is important to note that now, theoretically, a correlation between the measurement noise and process noise exists, since the DVL velocity measurement Equation (25) depends on the DVL partial raw data (with measurement noise) but also on the INS velocity solution Equation (24) (with process noise). Therefore:

(26) $$E\langle n_{w},{\bar{w}}^{T}\rangle \neq 0$$

Thus, the covariance term Equation (26), which is usually nullified in the EKF formulation, needs to be included. However, in our current analysis we neglect this term.

In order to calculate the corresponding measurement covariance matrix, we need to derive an expression for the constructed third beam measurement standard deviation (STD) $\sigma_{v3}$. To that end we use the error covariance matrix $p_{k-1}$ (last step of the fusing process), to define the velocity STD by:

(27) $$\sigma_{u}=\sqrt{P_{7,7}};\;\sigma_{v}=\sqrt{P_{8,8}}\;;\;\sigma_{w}=\sqrt{P_{9,9}}$$

Using Equation (24) we derive the expression for $\sigma_{v3}$:

(28) $$\sigma_{v3}=n_{f}\sqrt{{\left(b_{3,1}\sigma_{u}\right)}^{2}+{\left(b_{3,2}\sigma_{v}\right)}^{2}+{\left(b_{3,3}\sigma_{w}\right)}^{2}}$$

where $b_{i,j}=b_{i}(j)$ and $n_{f}$ is a factor to compensate for our assumption of neglecting Equation (26). Using the known values of for $\sigma_{v1}$ and $\sigma_{v2}$ and Equation (28), the DVL measurement noise covariance matrix is:

(29) $$R_{VB3D}=\left[\begin{array}{ccc} \sigma_{v1}^{2} & 0 & 0\\ 0 & \sigma_{v2}^{2} & 0\\ 0 & 0 & \sigma_{v3}^{2} \end{array}\right]\;W_{s}=R_{VB3D}^{-1}$$

From Equation (29) the velocity measurement noise covariance matrix is expressed via:

(30) $$R_{VB}={\left({A_{s}}^{T}W_{s}A_{s}\right)}^{-1}$$

In summary, in the proposed approach we calculate one velocity beam of the DVL via Equation (24). Combining the two beam measurement from the DVL, we solve the velocity vector of the vehicle using Equation (25).

### 3.2. Nullfying Sway Velocity

In this method we set the sway velocity to be zero, that is $v_{k}=0$. This assumption is reasonable for scenarios such as straight line trajectories, which in practice are the AUV trajectories for most of the operating time (although the AUV may be influenced by some disturbances that alter the straight line). Since we assume *v* = 0, only the other two velocity components (*u, w*) are to be calculated using the partial DVL data. Similar to the previous method outlined in Section 3.1, we assume that beams #3 and #4 are not available.

Let:

(31) $$A_{0}=\left[\begin{array}{cc} b_{1,1} & b_{1,3}\\ b_{2,1} & b_{2,3} \end{array}\right]$$

This matrix relates the beam velocities #1 and #2 (since beams #3 and #4 are not available) to the vehicle velocity (*u* and *w*). Thus, the relation between DVL beam velocities $y_{v1}$ and $y_{v2}$ to vehicle velocities is:

(32) $$\left[\begin{array}{c} y_{v1}\\ y_{v2} \end{array}\right]=A_{0}\left[\begin{array}{c} u\\ w \end{array}\right]$$

From Equation (32) the surge and heave velocities are:

(33) $$\left[\begin{array}{c} u_{k}\\ w_{k} \end{array}\right]=\left[A_{0}^{-1}\right]\left[\begin{array}{c} y_{v1}\\ y_{v2} \end{array}\right]$$

Note that the matrix $A_{0}$ is not singular, because of the independence of the beams direction vectors. The DVL measurement noise covariance matrix is then:

(34) $$R_{NSV2D}=\left[\begin{array}{cc} \sigma_{v1}^{2} & 0\\ 0 & \sigma_{v2}^{2} \end{array}\right]\;W_{0}=R_{NSV2D}^{-1}$$

and

(35) $$R_{0}={\left(A_{zero}^{T}W_{0}A_{zero}\right)}^{-1}$$

From Equation (35) we extract the velocity variances of the calculated vehicle velocity components (*u* and *w*). Therefore, the velocity measurement noise covariance matrix that is plugged into the navigation filter is:

(36) $$R_{NSV}=\left[\begin{array}{ccc} R_{0}[1,1] & 0 & 0\\ 0 & \varepsilon & 0\\ 0 & 0 & R_{0}[2,2] \end{array}\right]$$

where ε is a parameter that reflects the assumption of *v* = 0 and has a value close to zero. Equation (36) is used for practical considerations since the TAUV INS requires a complete velocity vector with its corresponding measurement covariance.

In summary, in the proposed approach we assume the sway velocity component is zero. Combining this assumption with the two beams measurements from the DVL, we solve the velocity vector of the vehicle using Equation (33).

### 3.3. Partial LC Fusing

This method utilizes the DVL setup configuration in order to calculate one component of the AUV velocity, *u* or *v*, depending on the active transducer order. Therefore, in this approach only one velocity component measurement is fused into the filter. As in the previous approaches, we assume that beams #3 and #4 are not available. When the DVL setup is in an “x” configuration, as in the TAUV, we can derive the following relations between the components of the DVL beam direction vectors based on Equation (13):

(37) $$\begin{array}{l} b_{1,1}=-b_{2,1}\\ b_{1,2}=b_{2,2}\\ b_{1,3}=b_{2,3} \end{array}$$

If the DVL setup is in a “+” configuration and the available beams are pointing in the same direction, the two components of the velocity vector—*u* and *w* or *v* and *w*—can be calculated. If the active beams are not pointing in the same direction (e.g., beam #1 is aimed toward the surge direction and beam #2 toward the sway direction) we cannot utilize any data from the DVL using this method. Here, (under the assumption that only beams #1 and #2 are available), we intend to solve the surge velocity *u*, utilizing the partial measurements from the DVL. Using Equation (37) we can express the velocity of beams #1 and #2 as:

(38) $$\begin{array}{l} b_{1,1}u+b_{1,2}v+b_{1,3}w=y_{v1}\\ -b_{1,1}u+b_{1,2}v+b_{1,3}w=y_{v2} \end{array}$$

Subtraction gives:

(39) $$2b_{1,1}u=y_{v1}-y_{v2}$$

The sway velocity is found using Equation (39):

(40) $$u_{k}=\frac{y_{v1}-y_{v2}}{2b_{1,1}}$$

where the corresponding measurement noise covariance is found using Equation (40):

(41) $$R_{u,PLCF}=\frac{\sigma_{v1}^{2}+\sigma_{v2}^{2}}{4b_{1,1}^{2}}$$

For practical considerations in the TAUV INS, the velocity measurement noise covariance matrix must have a structure of $R_{PLCF}\in {\mathbb{R}}^{3\times 3}$. So, the matrix that is passed to the filter is defined by:

(42) $$R_{PLCF}=\left[\begin{array}{ccc} R_{u,PLCF} & 0 & 0\\ 0 & R_{v,PLCF} & 0\\ 0 & 0 & R_{w,PLCF} \end{array}\right]$$

Yet, since only the surge velocity, u, is measured, variances $R_{v,PLCF}$ and $R_{w,PLCF}$, corresponding to sway and heave velocities, are:

(43) $$R_{v,PLCF}\to \infty ,\;R_{w,PLCF}\to \infty$$

To summarize, in the proposed approach we calculate the surge velocity component using the two beam measurements Equation (40). The filter ignores the two other velocity components (sway and heave) by setting their measurement variance to infinity. That is, in practice only one velocity component is used to aid the INS.

### 3.4. Virtual Heave Velocity

This method is an elaboration of the previous method derived in Section 3.3, where we extract the surge velocity *u* from the DVL raw data, while the DVL allows us access to two measured velocities. In order to utilize the two DVL measurements, we use the velocity prediction from the filter. Therefore, in this approach two vehicle velocity components are used to aid the INS: *u* is taken from Section 3.3, and *v* is calculated here. To that end, we sum the two equations from Equation (38) to derive the following expression:

(44) $$2b_{1,2}v+2b_{1,3}w=y_{v1}+y_{v2}$$

Thus, we have one equation, Equation (44), with two unknown velocity components, v and w. To calculate them we use the estimated velocity from the navigation filter. Assuming the AUV is travelling in a straight line, the heave velocity (w) estimation is probably more accurate due to the gravitation vector in heave (and even more so if the pressure sensor is active to measure the vehicle depth). Using Equation (44) and $w_{k-1}$ from the filter, the sway velocity is:

(45) $$v_{k}=\frac{y_{v1}+y_{v2}}{2b_{1,2}}-\frac{b_{1,3}}{b_{1,2}}{\hat{w}}_{k-1}$$

The corresponding velocity variance component is:

(46) $$R_{v,VHV}=\frac{\sigma_{v1}^{2}+\sigma_{v2}^{2}}{4b_{1,2}^{2}}-\frac{b_{1,3}^{2}}{b_{1,2}^{2}}{\sigma_{w}}^{2}$$

From Equations (41) and (46) the velocity measurement noise covariance matrix that is plugged into the navigation filter is given in Equation (47). As before, for practical considerations in the TAUV INS, the velocity measurement noise covariance matrix must have a structure of $R_{VHV}\in {\mathbb{R}}^{3\;\times \;3}$; thus we set $R_{w,VHV}\to \infty$, and the measurement noise covariance matrix is:

(47) $$R_{VHV}=\left[\begin{array}{ccc} R_{u,PLCF} & 0 & 0\\ 0 & R_{v,VHV} & 0\\ 0 & 0 & R_{w,VHV} \end{array}\right]$$

where $R_{u,PLCF}$ is defined in Equation (41) and $R_{v,VHV}$ is defined in Equation (46).

In summary, in the proposed approach we use the surge velocity component calculated as in Section 3.3. Using the estimated heave velocity component, the sway velocity component is solved via Equation (45). The filter ignores the other velocity component (heave) by setting its measurement variance to infinity. That is, in practice only two velocity components are used to aid the INS.

### 3.5. Summary of ELC Methods

Table 1 presents a summary of the four ELC methods. VB: virtual beam; NSV: nullifying sway velocity; PLCF: partial loosely coupled fusing; VHV: virtual heave velocity

### 3.6. ELC Implemenation

The DVL enables access to its raw data and its calculated velocity. In the case of partial measurements, the DVL cannot calculate the vehicle velocity, and only the partial raw data is available. From the raw data, we can calculate the vehicle velocity and the variance from all four approaches as presented in the previous sections. Instead of choosing which approach to use in the TAUV, in Equation (48) we employ a simple selector to pick the best vehicle velocity components out of the four approaches and use each derived corresponding velocity to aid the INS.

(48) $$\begin{array}{l} R_{u}=\min\{R_{u,\mathrm{VB}},R_{u,NSV},R_{u,PLCF},R_{u,VHV}\}\\ R_{v}=\min\{R_{v,\mathrm{VB}},R_{v,NSV},R_{v,PLCF},R_{v,VHV}\}\\ R_{w}=\min\{R_{w,\mathrm{VB}},R_{w,NSV},R_{w,PLCF},R_{w,VHV}\} \end{array}$$

The velocity measurement noise covariance matrix (with the corresponding velocity measurement) that goes to the TAUV INS is:

(49) $$R_{vel}=\left[\begin{array}{ccc} R_{u} & 0 & 0\\ 0 & R_{v} & 0\\ 0 & 0 & R_{w} \end{array}\right]$$

**Figure 7:**
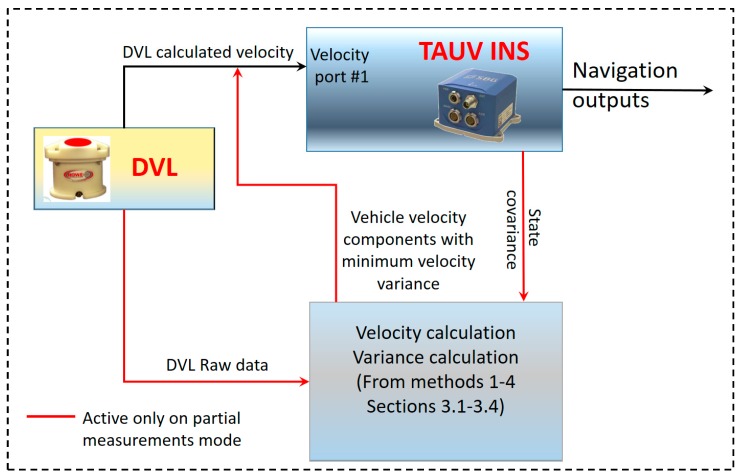
Block diagram for implementation of ELC approach in the TAUV navigation system.

Figure 7 shows the implementation of the ELC approach and LC approach in the TAUV. The red lines are relevant only in partial measurement scenarios.

## 4. Analysis and Results

### 4.1. Simulation Scenarios and Parameters

**Figure 8:**
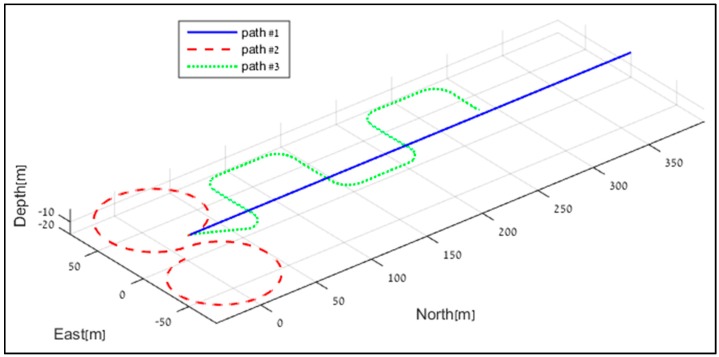
Simulation trajectories used to examine the ELC approaches.

In this section, we evaluate the ELC approach under three commonly used trajectories of the TAUV and other AUVs. The objective of using several trajectories is to evaluate the navigation performance of each method under different dynamic conditions. Figure 8 shows the chosen trajectories for the analysis. The first trajectory is a straight-line trajectory along the north direction while the AUV travels with constant speed and depth. This trajectory is chosen since in steady state the TAUV will, theoretically, travel in straight lines. The second trajectory follows a figure eight pattern. This trajectory contains permanent maneuvers and also a dive with constant velocity. The third trajectory is a classic seeking trajectory (lawn mower pattern) used for bottom survey. This trajectory begins with a dive at constant rate, and then in the searching phase constant depth is kept.

The simulation and navigation parameters used to produce the results in the paper are summarized in Table 2. The navigation parameters were chosen according to a literature survey [27] and the actual TAUV sensors specifications [16,26].

### 4.2. Simulation Results

The navigation performance obtained using Monte Carlo runs with the 6DOF simulation is presented in terms of the root mean square (RMS) errors of the velocity vector and the attitude error under the scenarios of trajectories #1, #2 and #3. The accelerometer and gyro biases are not presented since they behave as expected when fusing any other velocity measurements. That is, when the vehicle travels with constant velocity (trajectory #1) only the z-axis accelerometer bias and x and y gyro bias are observable. When maneuvering (trajectories #2 and #3) the estimation performance improves in terms of more observable error states.

We compare the performance of the ELC to the performance of the standalone INS (which is the case in the TAUV with partial DVL measurements scenario) and TC fusion, in order to test the effectiveness of the approach. We note that the analysis was made only for DVL/INS fusion. We expect that when using other available sensors on the TAUV (such as PS or magnetometer), the performance of the proposed approach will improve but this is not the subject of the paper.

In the next figures, the following notations are used: TC (tightly coupled; Section 2.1.3), VB (virtual beam; Section 3.1), NSV (nullifying sway velocity; Section 3.2), PLCF (partial loosely coupled fusing; Section 3.3), VHV (virtual heave velocity Section 3.4).

#### 4.2.1. Trajectory #1 Simulation Results

**Figure 9:**
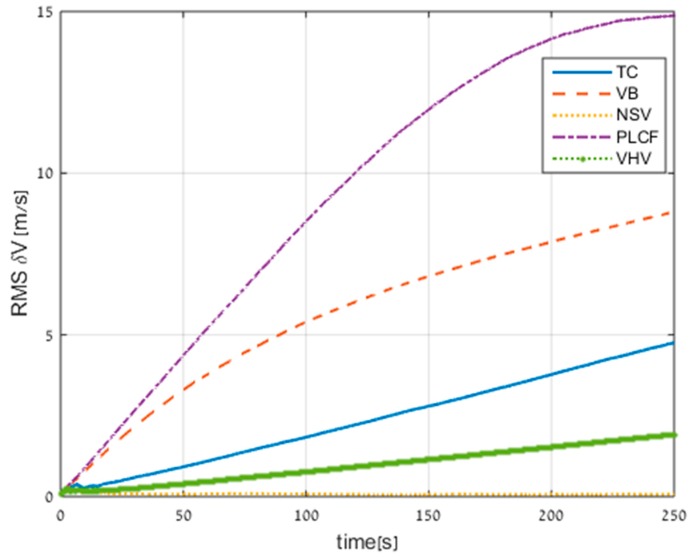
Root mean square (RMS) errors of velocity for all approaches obtained from trajectory #1. TC: tightly coupled.

The velocity RMS errors for all the methods (including TC) are presented in Figure 9. Not presented in the figure is the standalone INS performance, which is 34 m/s of error after 250 s.

All the proposed approaches improved the standalone INS performance; in particular, the NSV reduced the RMS velocity error to 0.05 m/s. In addition, the NSV approach and the VHV approach also performed better than the TC approach. The reason for this result is the assumption that the AUV does not have velocity in the sway direction, and in the case of a straight trajectory this assumption is reasonable. The PLCF obtained the worst performance but still improved the standalone INS solution by 131%.

**Figure 10:**
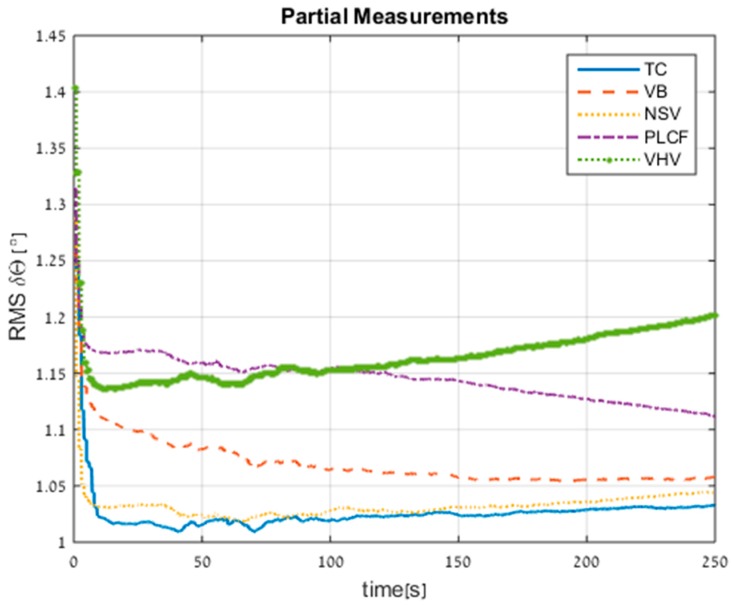
RMS errors of attitude for all approaches obtained from trajectory #1.

Figure 10 shows the RMS errors of the orientation misalignment. The standalone INS performance is 1.4° after 250 s. All proposed approaches improved the standalone INS; in particular the NSV and VB approaches achieved 33% improvement. The PLCF attitude error seemed to converge in time, due to the observation of the roll angle (in this method we use surge velocity aiding).

#### 4.2.2. Trajectory #2 Simulation Results

**Figure 11:**
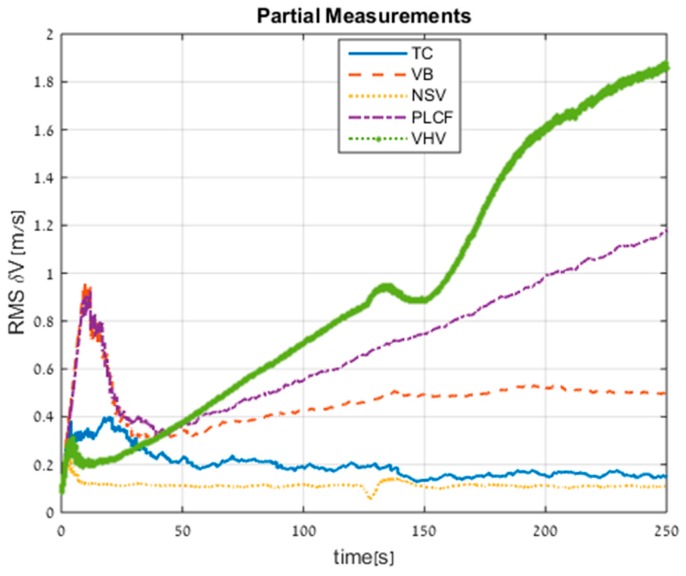
RMS errors of velocity for all approaches obtained from trajectory #2.

The velocity RMS errors for all the methods (including TC) are presented in Figure 11. Not presented in the figure is the standalone INS performance, which is 31.8 m/s of error after 250 s. All the proposed approaches improved the standalone INS; for example, the PLCF approach reduced the RMS velocity error to 1.2 m/s. In addition, the NSV approach also managed to perform better than the TC approach, but only with minor differences. Notice that all approaches, including TC, achieved better performance than in trajectory #1 due to the maneuvering of the AUV.

**Figure 12:**
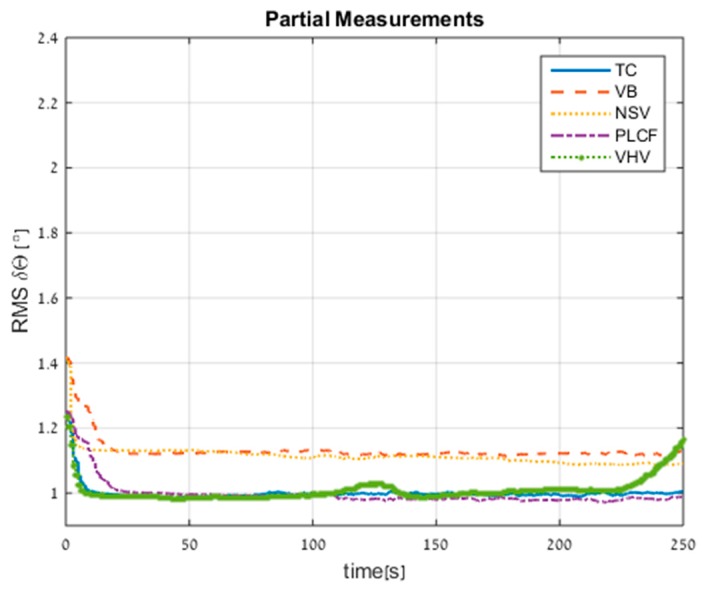
RMS errors of attitude for all approaches obtained from trajectory #2.

Figure 12 shows the RMS error of the attitude errors. The standalone INS performance is 1.26° after 250 s. As in the velocity RMS errors, all proposed approaches improved the standalone INS, in particularly the PLCF, which achieved a 26% improvement and the same performance as TC.

#### 4.2.3. Trajectory #3 Simulation Results

**Figure 13:**
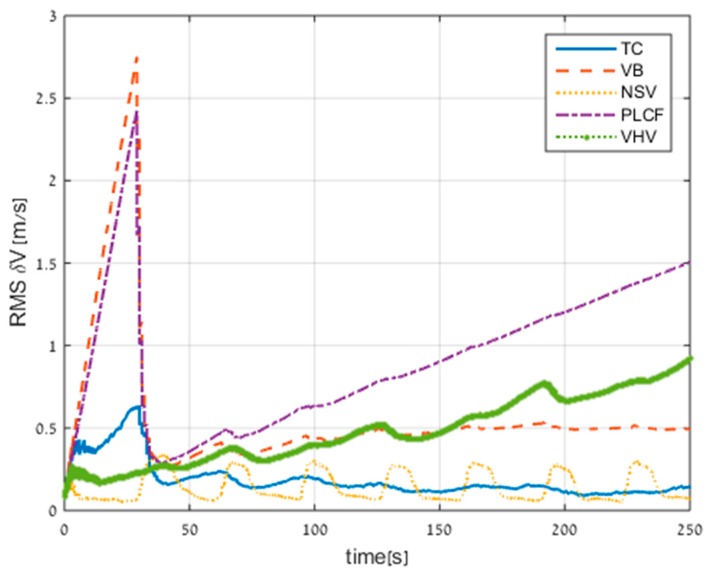
RMS errors of velocity for all approaches obtained from trajectory #3.

Figure 13 presents the velocity RMS errors of all ELC approaches and the TC approach. The standalone INS velocity error in this trajectory is 36.5 m/s after 250 s. As seen in Figure 10, all the proposed approaches improved the standalone INS, and the VB approach reduced the RMS velocity error to 0.5 m/s. The NSV approach managed to obtain similar performance to that of the TC, although its velocity error oscillates due to the change in the maneuvering direction. In the first 30 s, the velocity errors of methods VB and PLCF drift rapidly in time. The reason for this behavior is that the vehicle performs diving in that time period. After the TAUV completes the diving and reaches its desired depth, the velocity error of the VB approach does not drift in time.

**Figure 14:**
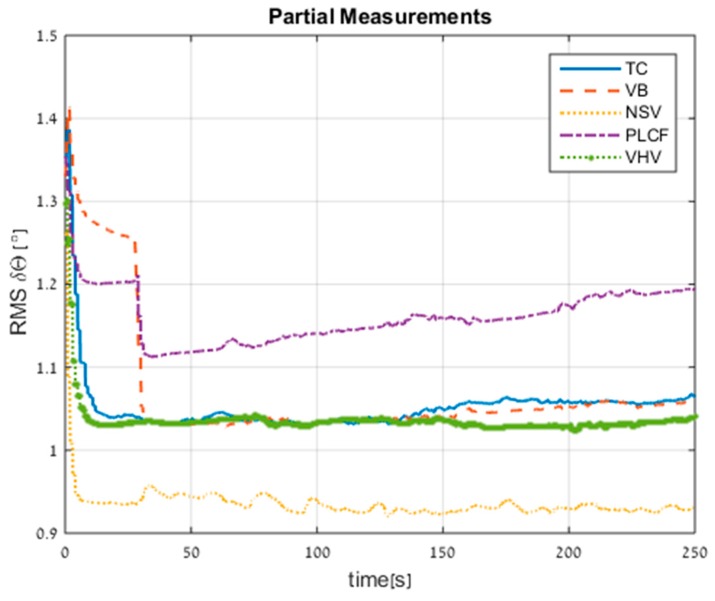
RMS errors of attitude for all approaches obtained from trajectory #3.

Figure 14 shows the RMS errors of the attitude. The standalone INS performance is 1.29° after 250 s. All proposed approaches improved the standalone INS; in particular, the NSV achieved 38% improvement and even achieved an improvement of 12% relative to the TC approach.

## 5. Conclusions

In the presented study the ELC approach for INS/DVL fusion with partial DVL measurements was investigated, applying four different methods. The TAUV simulation was developed in order to evaluate the ELC methods.

Results show that the presented approaches significantly improved the navigation performance of the TAUV in cases where only partial measurements from the DVL are available. In order to implement the suggested approach in the TAUV, only software modifications are needed.

## Figures and Tables

**Table 1:** ELC Methods.

Method	Assumtion	External Information	Calculations	Setting
VB (Section 3.1)	None	Last estimated velocity vector	Velocity vector Equation (24)	None
NSV (Section 3.2)	Zero sway velocity	None	Two velocity components Equation (33)	None
PLCF (Section 3.3)	None	None	One velocity component Equation (40)	Two velocity components with infinity variance
VHV (Section 3.4)	None	Last estimated heave velocity	Two velocity components Equations (40) and (45)	One velocity component with infinity variance

**Table 2:** TAUV 6DOF simulation and navigation parameters.

Parameter	Value
Time duration	250 (s)
AUV velocity	2 (m/s)
Accelerometer bias	0.5 (mg/h)
Gyro bias	3 (°/h)
Accelerometers noise	0.072 (m/s/√h)
Gyro noise	0.34 (°/√h)
Accelerometer bias random walk	$1\times 10^{-5}\;\left(m/s^{2}/\sqrt{s}\right)$
Gyro bias random walk	$2.8\times 10^{-5}\;$(°/s$/\sqrt{s}$)
Position initial error	north: 2 (m), east: 2 (m), height: 2 (m)
Velocity initial error	u: 0.05 (m/s) × v: 0.05 (m/s) × w: 0.05 (m/s)
Attitude initial error	Yaw: 1.14 (°) roll/pitch: 0.57 (°)
IMU rate	150 (Hz)
DVL rate	1 (Hz)
DVL noise	0.042 (m/s)
DVL bias	0.005 (m/s)
DVL bias random walk	$5\times 10^{-5}\;$(m/s/√s)
DVL scale factor	0.7 (%)
DVL scale factor random walk	$5\times 10^{-3}\;$(%/√s)
Magnometer noise	Yaw: 5.72 (°) × roll/pitch: 1.15 (°)
Pressure sensor noise	194 (Mpa)
Magnometer rate	0.5 (Hz)
Pressure sensor rate	0.25 (Hz)
